# Pelvines intraoperatives Neuromonitoring – Update und Pilotstudie zum Telementoring

**DOI:** 10.1055/a-2640-0212

**Published:** 2025-08-08

**Authors:** Werner Kneist, Daniel Wilhelm Kauff, Tobias Huber, Jonas Friedrich Schiemer, Markus Paschold

**Affiliations:** 1 9143 Chirurgische Klinik 1, Klinik für Allgemein-, Viszeral- und Thoraxchirurgie, Klinikum Darmstadt, Darmstadt, Deutschland; 2 155803 Klinik für Allgemein-, Viszeral- und Gefäßchirurgie mit Unfallchirurgie Nagold, Klinikverbund Südwest GmbH, Sindelfingen, Deutschland; 3 39068 Klinik für Allgemein-, Viszeral- und Transplantationschirurgie, Universitätsmedizin der Johannes Gutenberg-Universität, Mainz, Deutschland; 4 72204 Klinik für Allgemein- und Viszeralchirurgie, St Josefs-Hospital Wiesbaden, Wiesbaden, Deutschland; 5 502932 Klinik für Allgemein- und Viszeralchirurgie, Krankenhaus St. Marienwörth, Bad Kreuznach, Deutschland

**Keywords:** pelvines Neuromonitoring, Rektumkarzinom, Telemonitoring, funktionelle Ergebnisse, chirurgische Weiterbildung, pelvic neuromonitoring, rectal cancer, telementoring, functional outcomes, surgical education

## Abstract

Das pelvine intraoperative Neuromonitoring (pIONM) gewinnt zunehmend an Bedeutung als Instrument zur Verbesserung funktioneller Ergebnisse nach Rektumchirurgie. Diese Arbeit fasst aktuelle Erkenntnisse zum pIONM zusammen, darunter Ergebnisse der multizentrischen, randomisierten NEUROS-Studie, die signifikante Vorteile für urogenitale und ano-(neo-)rektale Funktionen belegt. Zudem wird die Rolle des Telementorings als Schlüssel für die standardisierte Einführung des robotisch assistierten pIONM untersucht. Eine Pilotserie demonstriert bei drei Operationen den geringen kognitiven Aufwand für Chirurgen während des telemedizinisch unterstützten Monitorings, gemessen mittels NASA-TLX-Fragebogen (TLX-Score Spannweite von 5.0 bis 24.3). Die Ergebnisse unterstreichen das Potenzial des pIONM, Nervenverletzungen zu vermeiden und die Lebensqualität von Patienten zu steigern, während Telementoring die Verbreitung der Methode erleichtern könnte.

## Abstract (en)

Pelvic intraoperative neuromonitoring (pIONM) is emerging as a key tool to enhance functional outcomes following rectal cancer surgery. This paper synthesizes current evidence on pIONM, including findings from the multicenter, randomized NEUROS trial, which demonstrated significant benefits for urinary, sexual, and ano-(neo-)rectal functions. Additionally, the role of telementoring as a catalyst for standardized robotic assisted pIONM adoption is explored. Three case studies illustrate the low cognitive burden on surgeons during telemedicine-assisted monitoring, assessed via the NASA-TLX questionnaire (TLX-score varied from 5.0 to 24.3). The results highlight pIONM’s potential to prevent nerve damage and improve patient quality of life, with telementoring poised to facilitate broader implementation.

## Einleitung

Nach totaler mesorektaler Exzision (TME) bei Patienten mit Rektumkarzinom werden Störungen der Miktion (> 30%), der sexuellen Funktion (> 50%) und der Defäkation (37–90%) beschrieben 1 2 . Von neurogen verursachten Störungen ist bei einem Drittel der Patienten mit postoperativ neu aufgetretenen Miktionsstörungen auszugehen. Ähnlich hoch dürfte der entsprechende Anteil für das tiefe anteriore Resektionssyndrom (LARS) und weit höher bei sexuellen Funktionsstörungen sein. Das pelvine intraoperative Neuromonitoring (pIONM) wird aktuell neben der robotisch assistierten Präzisionschirurgie als vielversprechende intraoperative Möglichkeit zur Vermeidung urogenitaler und anoneorektaler Funktionsstörungen gewertet 3 4 . Das neuroprotektive Assistenzsystem wird sogar zu den 10 wichtigsten Forschungsschwerpunkten in Bezug auf die Behandlung von Patienten mit kolorektalem Karzinom gezählt 5 . Es hat die präklinischen Phasen sowie die Ideen- und Entwicklungsstufen iterativ erfolgreich durchlaufen. Da aber die große Mehrheit spezialisierter Rektumchirurgen und -chirurginnen mit der Technik nicht vertraut sind und Argumente wie „aufwendig“ und „ressourcenintensiv“ bestehen, ist die weitere Beschäftigung mit dem Thema sinnvoll. In der vorliegenden Arbeit sollen aktuell publizierte Ergebnisse zum pIONM zusammengefasst werden. Da nun auch die Schnittstelle zur Robotik gut etabliert ist 6 7 8 9 10 11 12 , wäre eine effektive, kollaborative telemedizinische Einführung des Neuromonitorings möglich und innovativ. In 3 Fällen untersuchten wir daher unseren Aufwand für ein benutzerfreundliches Telementoring.

## Methode

**Abb. 1:**
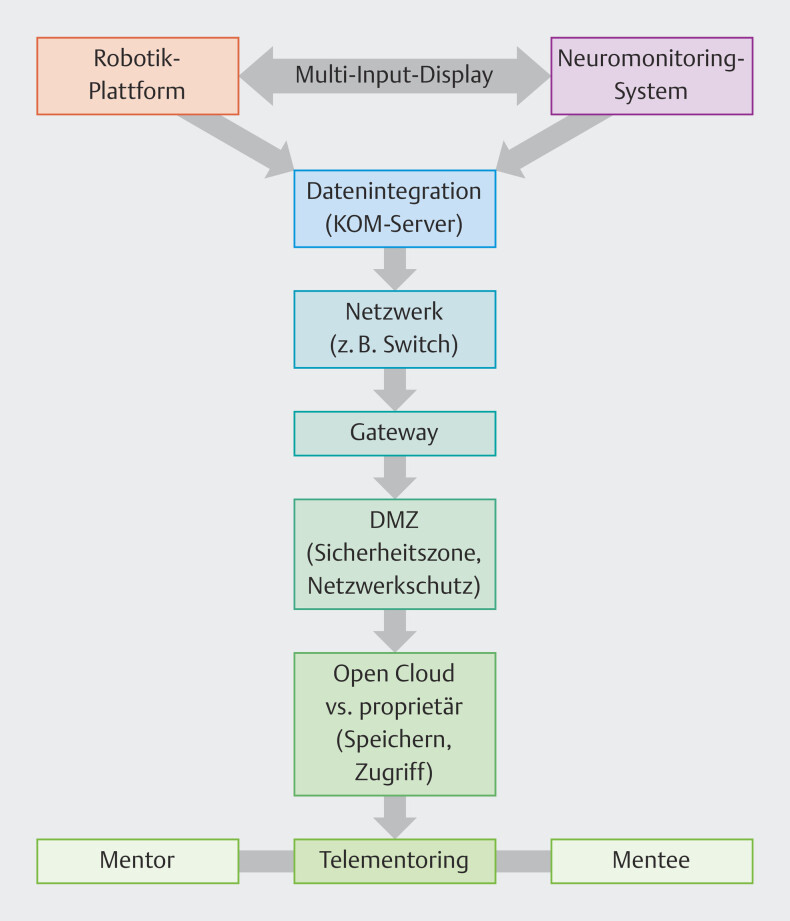
Telemonitoring-Komponenten-KOM-Server: Kommunikationsserver; zentrale Schnittstelle für sicheren, standardisierten herstellerunabhängigen Datenaustausch; Switch: zentrales Netzwerkgerät zur sicheren Weiterleitung von Datenpaketen in einem lokalen Netzwerk (LAN); vernetzter OP-Roboter, Neuromonitoring, Intuitive Hub, KOM-Server und Krankenhausinformationssystem; Echtzeit-Datenverkehr durch Priorisierung; Segmentierung ermöglicht isolierte, sichere virtuelle Netze (VLANs); Gateway: Schlüsselkomponente, die unterschiedliche Netzwerke, Protokolle, Formate miteinander verbindet (Brücke zwischen inkompatiblen Systemen). DMZ: demilitarisierte Zone; isoliertes Netzwerksegment; IT-Sicherheitsstandard (Pufferzone) zwischen einem vertrauenswürdigen (internen) und einem unsicheren (externen Netzwerk); Open Cloud: keine Lizenzgebühren, flexibel anpassbar, mehr IT-Aufwand, transparent proprietäre Lösung: herstellergesteuert, hohe Benutzerfreundlichkeit, kostenintensiv, garantierter Support (z. B. Intuitive Hub).

Die wichtigsten Ergebnisse und Empfehlungen zum pIONM aus systematischen Reviews 13 14 und der ersten kontrollierten randomisierten Studie 15 werden zusammengefasst und medizintechnische Aspekte kurz beleuchtet. Zusätzlich analysierten wir erste telemedizinische Ergebnisse für eine effektive und sichere Einführung des robotisch geführten pIONM. Wir nutzten den Intuitive Hub 16 . Vorab zeigten sich Plattform und Vernetzung performant; die Themen Datenschutz, Informationssicherheit sowie Betriebsrat waren geklärt und aus informations-, telekommunikations- und medizintechnischer Sicht bestanden keine Bedenken ( Abb. 1 ). Die Teilnahme von 3 Patienten an unserer Telemonitoring-Pilotstudie war freiwillig und ergänzte die Routineversorgung mit robotisch assistierter TME und pIONM am Viszeralonkologischen Zentrum der Klinikum Darmstadt GmbH. Vier mit dem pIONM sehr vertraute Kollegen DWK, MP, TH und JFS wurden für die Dauer des pIONM bei 2 Operationen im Remote-Modus zugeschaltet. Bei einer 3. Operation wurde das pIONM deutschlandweit gestreamt 17 .

Die Erfassung der kognitiven Belastung während des pIONM erfolgte mit dem validierten NASA-TLX-Fragebogen (Subskalen: mentale/physische/zeitliche Anforderungen, empfundene Leistung, Anstrengung und Frustration). Die unmittelbar postoperativ erhobenen Einzelbewertungen der Chirurgen wurden durch die offizielle NASA-TLX-iOS-App (Apple Inc.) berechnet 18 . Es wurden keine personenbezogenen Daten an die von NASA-TLX genutzten Datenserver übermittelt.

Referenzwerte aus der Literatur zeigen, dass der TLX-Score übergreifend Mittelwerte zwischen 35 (Freizeitaktivitäten) und 56 (schwere körperliche Arbeit) aufweist, wobei eine Einordnung anhand publizierter Perzentilen möglich ist (10. Perzentile: 26; 50. Perzentile: 43; 90. Perzentile: 57) 19 .

## Aktuelle Literaturübersichten

Zwei Reviews befassen sich direkt mit den Ergebnissen des pIONM. Nebenwirkungen oder Komplikationen im Zusammenhang mit der Methode wurden nicht beschrieben. Insbesondere bei der TME war der Einfluss auf die postoperativen funktionellen Ergebnisse positiv. Die Metaanalyse von Samara et al. 13 zeigte die signifikanten Verbesserungen der Miktions- und Sexualfunktion (3–20% vs. 40–54%; 38–60% vs. 68–91%) und die Reduktion der Stuhlinkontinenz (3–21% vs. 35–50%) bei intraoperativer bilateraler Kontrolle der Nervenschonung. Die Autoren wiesen auf die Heterogenität der Daten (von 7 prospektiven und 2 retrospektiven Studien) und das Fehlen randomisierter Studien hin. Das systematische Review von O’Connor et al. 14 berücksichtigte 20 Studien mit 686 Patienten und bewertete die diagnostische Genauigkeit des pIONM. Es wurde eine hohe Treffsicherheit bei der Vorhersage von anoneorektalen Funktionsstörungen (Sensitivität 1,00 [95%-KI: 0,03–1,00], Spezifität 0,98 [95%-KI: 0,91–0,99]) und Harnblasenfunktionsstörungen (Sensitivität 1,00 [95%-KI: 0,03–1,00], Spezifität 0,99 [95%-KI: 0,92–0,99]) attestiert, insbesondere, wenn 2-dimensional sowohl EMG des internen analen Sphinkters als auch die Harnblasenmanometrie als Erfolgsmessung verwendet wurden. Daraufhin wurde postuliert, dass ein pIONM dazu beitragen kann, Patienten mit einem hohen Risiko für funktionelle Störungen frühzeitig zu identifizieren und präventive Maßnahmen gezielt einzuleiten (z. B. Harnableitung, Beckenbodentraining, transanale Irrigation, Pharmakotherapie, Psychosomatik, Sakralnervenmodulation).

## NEUROS-Studie

Die im Jahr 2023 veröffentlichte multizentrische, randomisierte, kontrollierte klinische Studie untersuchte, ob mithilfe eines intraoperativ eingesetzten NEUROmonitoring Systems (NEUROS) die urogenitalen und ano-(neo-)rektalen funktionellen Ergebnisse bei Patienten mit totaler mesorektaler Exzision (TME) verbessert werden können 15 . In die 2-armige Studie wurden zwischen Februar 2013 und Januar 2017 in 8 Zentren 189 Patienten mit Rektumkarzinom eingeschlossen (Interventions-Gruppe: TME mit intraoperativem Neuromonitoring [n = 90]; Kontrollgruppe: TME ohne Neuromonitoring [n = 99]).

Der primäre Endpunkt war die relevante Verschlechterung der Harnblasenfunktion 12 Monate nach der Operation, gemessen anhand des International Prostate Symptom Score. Sekundäre Endpunkte umfassten sexuelle und anoneorektale Funktionsergebnisse, Sicherheit und die Qualität der TME. In 85% der Fälle wurde minimalinvasiv operiert und in 43% erfolgte nach makroskopischer Einschätzung die Schonung der autonomen Beckennerven; signifikante Gruppenunterschiede bestanden nicht.

Es wurde ein CE-zertifiziertes Neuromonitoring-System verwendet (Inomed Medizintechnik GmbH, Emmendingen, Deutschland). Die Nervenstimulation erfolgte über eine handgeführte bipolare Mikrogabelsonde. Das Stimulationsergebnis wurde mittels kontinuierlicher elektromyografischer Ableitung des inneren Analsphinkters unter gleichzeitiger Manometrie der Harnblase erfasst. Bilateral wurden 4 definierte Schritte eingehalten. Die initialen Stimulationen erfolgten während der posterioren/posterolateralen mesorektalen Dissektion, um die Nn. splanchnici pelvici zu identifizieren. Während der lateralen Dissektion wurde ein Neuromapping entlang der Beckenseitenwand durchgeführt, um weiteres potenzielles Nervengewebe und den Plexus hypogastricus inferior zu identifizieren. Bei der anterolateralen mesorektalen Dissektion wurde die extrinsische Nervenversorgung des M. sphincter ani internus getestet. Nach der Resektion des Präparats wurde die autonome Innervation erneut mittels bilateralem Neuromapping überprüft.

NEUROS liefert erstmals hochwertige Evidenz, die die Vorteile des pIONM bei der TME für Patienten mit Rektumkarzinom belegt ( Tab. 1 ). Es gab keine signifikanten Unterschiede zwischen den Gruppen in Bezug auf TME-Qualität, Operationszeiten, intraoperative Komplikationen oder postoperative Mortalität. Schwerwiegende unerwünschte Ereignisse (SAEs) waren gleich verteilt und nicht auf das pIONM zurückzuführen.

**Table:** **Tab. 1** Hauptergebnisse der NEUROS-Studie 15 .

Ergebnisparameter	Interventionsgruppe (mit pIONM)	Kontrollgruppe (ohne pIONM)	p-Wert
Patientenzahl (ITT-Analyse)	82	89	–
Verschlechterung Harnblasenfunktion (Δ IPSS ≥ 5 Punkte)	6 (8%)	16 (19%)	0,038
sexuelle Funktion Männer (IIEF, Mittelwert ± SD)	40,3 ± 25	25,0 ± 19	0,012
sexuelle Funktion Frauen (FSFI < 26,6)	40%	70%	0,040
Stuhlinkontinenz (Wexner-Score, Mittelwert ± SD)	5,5 ± 4,5	7,9 ± 5,6	0,011
fraktionierte Entleerung (Symptom vorhanden)	56%	75%	0,021
pIONM: pelvines intraoperatives Neuromonitoring; IPSS: International Prostate Symptom Score; IIEF: International Index of Erectile Function; FSFI: Female Sexual Function Index			

Die Ergebnisse sprechen für die Einführung als Standardverfahren, um die postoperative Lebensqualität der Patienten zu verbessern. Register zur Erfassung der „Real-World-Daten“ sind wünschenswert und pIONM-Trainingsmodalitäten zu klären. Zukünftige kontrollierte Studien könnten sich dem nervenschonenden Potenzial verschiedener Dissektionstechniken und der Wirksamkeit frühpräventiver Maßnahmen widmen, um somit die Raten neurogener Dysfunktionen nach TME weiter zu reduzieren. Zunehmende Standardisierung, Digitalisierung und medizintechnische Weiterentwicklungen sind damit zu verbinden.

## Stand der Medizintechnik

**Abb. 2:**
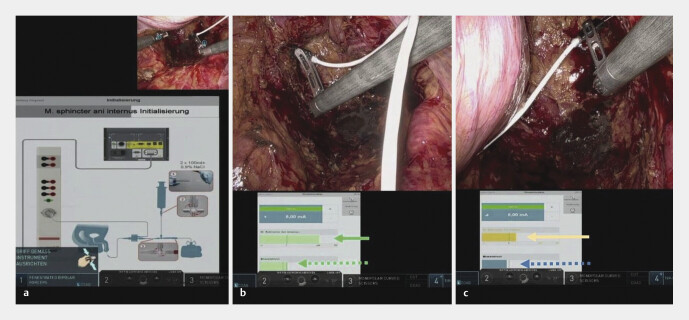
Abschließendes Neuromapping nach robotischer tiefer anteriorer (Diskontinuitäts-)Resektion mit totaler mesorektaler Exzision bei einem 89-jährigen Patienten mit stenosierendem Karzinom des mittleren Rektumdrittels (Rekatheterisierung bei postoperativer Blasenentleerungsstörung mit Restharnerhöhung). **a** Selbstcheck des Neuromonitoringsystems. **b** Rechtsseitige Neurostimulation am Plexus hypogastricus inferior mit positivem (grünem) Balkenausschlag für intakte Innervation sowohl des internen analen Schließmuskels (durchgezogener Pfeil) und der Harnblase (gestrichelter Pfeil). **c** Linksseitige Neurostimulation am Plexus hypogastricus inferior mit grenzwertigem (gelbem) Balkenausschlag für unsichere Innervation des internen analen Schließmuskels (durchgezogener Pfeil) und negativem Ergebnis (blauer Balken) an der Harnblase (gestrichelter Pfeil).

Nach erfolgreicher Translation sind Systeme zum pIONM bei laparoskopischer, transanaler und robotischer Operation sowie vernetzt im hybriden OP-Saal verwendbar 12 . Semiautomatisierte Neuromonitoring-Systeme alarmieren akustisch und visuell bei Signalveränderungen. Die Interpretation des intraoperativen Sphinkter-EMG und der Blasenmanometrie wird entsprechend unterstützt ( Abb. 2 ). Systemeigene Online-Signalverarbeitungs- und Artefaktunterdrückungsprogramme basieren auf Referenz- und Schwellenwerten, die klinisch evaluiert und anschließend integriert wurden. Mit einem Prototyp (Dr. Langer Medical GmbH, Waldkirch, Deutschland) konnte kürzlich bei 93,3% der operierten Patienten (n = 30) während der Rektumresektion eine stimulationsinduzierte Impedanzdifferenz an den pelvinen Erfolgsorganen beobachtet werden, die mit der postoperativen Funktion korrelierte. Eine automatisierte Signalinterpretation unterschied positive von negativen Stimulationsantworten und Artefakten mit einer Sensitivität von 96,3% und einer Spezifität von 91,2% und deutete damit das Potenzial einer bioimpedanzbasierten intraoperativen Nervenüberwachung als ebenfalls vielversprechende Technologie an 20 21 . Trotz medizintechnischer Fortschritte fehlt es weiterhin an DRG-Vergütungsstrukturen, was die flächendeckende Etablierung des pIONM erschwert. Durch die im Rahmen des Krankenhausversorgungsverbesserungsgesetzes einzuführende Leistungsgruppe „Tiefe Rektumresektion“ könnte sich das ändern.

## Training

Das intraoperative Neuromonitoring ist nur mit einem gut geschulten Team sinnvoll. Im Operationssaal soll zwischen echten und artifiziellen Stimulationsreaktionen unterschieden und ggf. geeignete Maßnahmen zur Identifizierung und Behebung von Problemen ergriffen werden (Troubleshooting). Für NEUROS 15 war ein umfassendes Training organisiert: 2 interprofessionelle Workshops für Mitarbeiter der Medizintechnik, 6 Anwenderworkshops, 8 externe DGAV-Workshops mit pIONM bei TME, firmenseitig dokumentiertes Techniktraining und klinische OP-Check-ups für alle teilnehmende Zentren. In der Rekrutierungsphase erfolgten On-Demand Case Visits vor Ort und Case Observations am Referenzzentrum in Mainz. Die hohe Treffsicherheit der Methode war bereits nachgewiesen und die Operateure der teilnehmenden zertifizierten Darmkrebszentren waren spezialisiert. Im Ergebnis gab es keine signifikanten Differenzen zwischen den Zentren der NEUROS-Studie. Auf Publikationen, Instruktionsmaterial und auf Videos zum pIONM kann zurückgegriffen werden.

## Telementoring

Bisher werden objektive Daten zur Schwierigkeit oder Leichtigkeit eines innovativen chirurgischen Verfahrens und entsprechende Details zu erforderlichen Trainings nur sehr selten erfasst (2–4%) 22 . Sicherlich sollte der Lehr- und Lernaufwand für eine intermittierende Assistenz von nur wenigen Minuten (z. B. pIONM, ICG oder Ultraschall) deutlich geringer sein 15 23 24 als für ein Curriculum komplexer kolorektaler Eingriffe 25 26 .

**Abb. 3:**
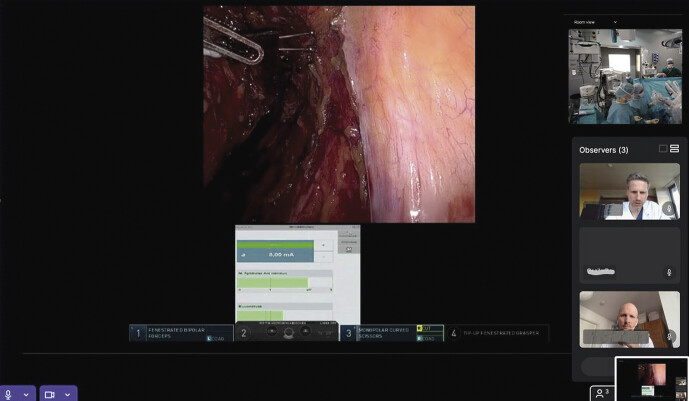
Livestreaming via Intuitive Hub. Rechtsseitige Neurostimulation am Plexus hypogastricus inferior während der mesorektalen Dissektion. Positive EMG- und Manometriesignale (Fall 1). Multiple Ansichten im Monitordisplay des Remote-Teams.

In einem Snap-Shot-Audit mit moderner Telekommunikationstechnologie können wir bei 2 TMEs zeigen, dass die Arbeitsbelastung für eine Case Observation sowohl für den Remote- als auch für den Vor-Ort-Chirurgen sehr gering ist ( Abb. 3 ). Telementoring via Intuitive Hub ermöglichte in 2 Fällen 4 Remote-Chirurgen die Echtzeitbeobachtung des pIONM innerhalb von 20 min. Die NASA-TLX-Bewertungen ergaben geringe Arbeitsbelastungen (Gesamt-TLX: 8,3 und 17,0 für Operateure; 7,7–11,7 für Remote-Team). Im 3. Fall der Pilotserie ( Abb. 2 ) wurde das abschließende Neuromapping nach TME gestreamt und erläutert (7 min). Mehr als 100 Chirurginnen und Chirurgen, die bei einer akkreditierten Weiterbildungsveranstaltung teilnahmen, waren zugeschaltet 17 .

In einem Benchmark-Vergleich mit 556 Studien 19 überschritt der TLX-Score bei keiner unserer Bewertungen das 10. Perzentil. Zudem lag der von uns erfasste Aufwand für das pIONM unter den angegebenen Mittelwerten aus 87 Studien mit Bewertungen anderer Prozeduren aus dem Gesundheitswesen ( Tab. 2 ).

**Table:** **Tab. 2** Gesamtaufwand (Task-Load-Indizes [TLX]) und Subskalenmuster für das Telementoring per Intuitive Hub 16 beim pelvinen intraoperativen Neuromonitoring während tiefer anteriorer Resektion mit totaler mesorektaler Exzision in 3 Fällen im Vergleich mit publizierten TLX-Referenzwerten für andere Prozeduren im Gesundheitssektor 19 .

	Fall 1			Fall 2			Fall 3	
NASA TLX	On-Site WK	Remote DWK	Remote JS	On-Site WK	Remote TH	Remote MP	On-Site WK	Referenzwert*
Gesamtaufwand	8,3	7,7	11,7	17,0	5,0	9,7	24,3	45 ± 16
mental	10	20	15	10	20	15	20	52 ± 19
physisch	5	0	5	10	0	5	10	36 ± 18
zeitlich	15	0	20	15	0	15	35	46 ± 18
empfundene Leistung	5	10	10	20	5	5	25	44 ± 22
Anstrengung	10	10	15	15	5	5	20	51 ± 17
Frustration	5	5	5	20	0	15	25	39 ± 16
NASA: National Aeronautics and Space Administration; TLX: Task Load Index; * NASA TLX Mittelwert ± Standardabweichung (n = 87 Studien aus dem Gesundheitssektor)								

Um inhärenten Einschränkungen und potenziellen Risiken entgegenzuwirken, sollte eine Checkliste für das Telementoring kolorektaler Eingriffe beachtet werden 27 . Das Team ist auch bez. der Telekommunikation umfassend zu schulen. Außerdem soll dem Erstanwender bei chirurgischen Problemen oder Telekommunikationsausfällen ein Backup zur Verfügung gestellt werden, um eine Prozedur (z. B. das pIONM) dennoch durchführen zu können.

Da eine moderne Art und Weise der Wissensvermittlung in der Chirurgie als Conditio sine qua non für eine auch didaktisch neu zu gestaltende Verbundweiterbildung eingefordert wird 28 , hat die „operative Telemedizin“ gute Chancen, gefördert zu werden. Operationssäle akademischer Lehrkrankenhäuser und Universitätskliniken müssen ohnehin als sog. „KRITIS-Häuser“ bereits mit sicherer, hochverfügbarer Informationstechnik (Hard- und Software, Netzwerksysteme, Desktop-Computern, digitalen Videoaufzeichnungssystemen mit MIC- bzw. Roboterschnittstellen) ausgestattet sein 29 . Für Telementoring-Plattformen sind die Kosten anbieter-, anwender- und anforderungsspezifisch (Hub-and-Spoke-Modelle/Telechirurgie/OP-Streaming/Lehre/Kollaboration …) sehr variabel. Für den von uns verwendeten Intuitive Hub inkl. Implementierung und Service kann mit Investitionen im mittleren 5-stelligen Bereich gerechnet werden.

## Fazit

Das pelvine intraoperative Neuromonitoring verbessert, urogenitale und ano-(neo-)rektale Funktionen nach Rektumoperationen signifikant, indem es Nervenverletzungen vermeidet und als diagnostisches Tool eingesetzt wird. NEUROS liefert erstmals hochwertige Evidenz aus einer randomisierten, kontrollierten Studie, die die Vorteile des intraoperativen Neuromonitorings bei der TME für Patienten mit Rektumkarzinom belegt. Die breite Implementierung wird durch fehlende Vertrautheit vieler Chirurgen mit der Methode gehemmt. Hier zeigt Telementoring als innovativer Ansatz großes Potenzial (geringe kognitive Belastung, Ressourcenschonung durch verkürzte Lernkurven, Remote-Schulungen und Troubleshooting). Aktuelle Medizin-, Informations- und Telekommunikationstechnik (z. B. automatisierte Signalanalyse, Intuitive Hub) unterstützen die standardisierte Anwendung innerhalb zukünftiger RCTs und Registerstudien (Stadium 4 des IDEAL Frameworks).
